# Mr. Swift's Cases of Dropsy

**Published:** 1801-04

**Authors:** J. S. Swift

**Affiliations:** Surgeon, Royal Navy. Jersey


### *To the Editors of the Medical and Phyfical Journal.*

Gentlemen,

THE following are two cafes of dropfy, cured by Digitalis; if you think them worthy of a page in your Journal, you will oblige me by inferting them.

I remain, yours, &c.

J. S. SWIFT, Surgeon, Royal Navy.

*Jersey, Feb.* 18, 1801.

---

December 4, 1800, I was requefted to fee Mrs. B. at Hardway, near Portfmouth, aged 52, who had been afflicted with afcites nearly four years. Symptoms were, a fluctuating tenfe abdomen, an irregular quick pulfe, continual thirft, paucity of urine and pale, with impeded refpiration: I gave her the following mixture, viz. ℞. Inf. Digital. ℥viij. fpt. æther. nitros. ℥fs. M. fumat ℥i. ter die.

5th. Complains of a little griping, otherwife much the fame. Rep. Mist. add. Tinct. Card. Comp. ʒiij.

6th,

6th. Bowels easy; voided three pints of urine in the last twenty-six hours. Having very little rest by night, I gave her an anodyne, (which was repeated occasionally during the cure). Rep. Mist. ut heri.

7th. Much as yesterday. Rep. Mist.

9th. Upon an average, she voids a quart of urine every ten hours; breath more free. Rep. Mist.

14th. Pulse more regular, swelling considerably diminished. Rep. Mist.

16th. Being costive, she took a laxative, and repeated the mixture, with a nourishing diet, until January 10th, 1801, when her body was reduced to its natural size, and every other symptom of ascites gone. She then took the Cort. Peruv. et Pulv. Aromat. until the 30th following, when she found herself quite recovered.

January 2, 1801, I saw T. Mason of Elson, near Portsmouth, aged seven, who had oedematous swelling of the head, body, &c. very little urine, and pale, continual thirst, and every other symptom of anasarca, was perfectly cured by the same treatment as Mrs. B's, in the space of six weeks.

